# Effects of Olfactory Learning Context Reactivation During Sleep on Training Success in a Double‐Blind Randomized Controlled Sensorimotor Rhythm Neurofeedback Protocol: A Pilot Study

**DOI:** 10.1111/psyp.70154

**Published:** 2025-10-08

**Authors:** Julia Lechinger, Catarina Newe, Svenja Nadler, Marlene Schubert, Hong‐Viet V. Ngo, Robert Göder, Alexander Prehn‐Kristensen

**Affiliations:** ^1^ Centre for Integrative Psychiatry, University Hospital Schleswig‐Holstein Kiel Germany; ^2^ Institute of Child and Adolescent Psychiatry, Centre for Integrative Psychiatry University Hospital Schleswig‐Holstein Kiel Germany; ^3^ Clinical Psychology and Psychotherapy, Department of Psychology Christian‐Albrechts University Kiel Germany; ^4^ Department of Psychology University of Essex Colchester UK; ^5^ Department of Psychology, Faculty of Human Sciences Medical School Hamburg Hamburg Germany

**Keywords:** alpha frequency, cue‐dependent memory reactivation, declarative memory, neurofeedback training, polysomnography, sleep spindles

## Abstract

Neurofeedback training (NFT) is used to modulate brain activity for therapeutic purposes in different patient populations. However, tested training protocols vary in several aspects, and the results of therapeutic benefit have been heterogeneous. The aim of the current study was to compare a SMR against a random frequency protocol and potentially strengthen NFT effects via olfactory context reactivation during sleep in a compact training protocol. 49 participants (mean age: 24.7 years; 29 women) without any neurological or psychiatric disorder were randomly (double‐blind) assigned to three groups: sensorimotor rhythm (SMR) with or without context reactivation, or random frequencies with reactivation during sleep. Within 2 weeks, participants underwent eight training sessions (12 × 2 min training intervals) and slept three nights in the sleep lab. NFT sessions were scheduled every day or every other day for practical reasons. Nights two and three were used for reactivation. SMR training success was assessed at baseline, post treatment, and follow‐up after 10 months. Neurofeedback was provided as pixelated video, which became clearer with better training performance. Although SMR amplitude did not significantly increase, NFT training seemed to have an effect on alpha amplitude over the course of training (*p* = 0.08), and both SMR groups showed by tendency improved objective sleep (e.g., higher sleep efficiency in night 2 vs. baseline night, p_corr_ = 0.07). Reactivation did not immediately affect performance or EEG response. However, during the follow‐up session, NFT performance was highest in the SMR + reactivation condition (SMR + *R* > SMR‐R, p_corr_ = 0.02), which could indicate a stabilizing effect of reactivation during sleep. Our data again suggest that (SMR) NFT could foster general relaxation and sleep. Concerning context reactivation, we did, however, only find limited evidence for a potential benefit. Future studies could add more reactivation nights or potentially utilize other forms of context reactivation.

## Impact Statement

Neurofeedback training (NFT) has shown promising effects in several clinical fields. There is still ongoing debate, however, whether the effects specifically depend on the trained frequency or the setting/method in general. Our aim was to compare both a SMR and random frequency protocol and test whether effects could be enhanced by olfactory reactivation of trainings context during sleep. We found specific effect of SMR NFT on sleep as well as a potential stabilizing effect of context reactivation.

## 1 Introduction

Neurofeedback training (NFT) is a non‐invasive, computerized method based on electroencephalography (EEG) that allows participants to gain voluntary control over their brain functions. NFT uses real‐time displays of EEG activity while training success is met with positive reinforcement, typically through visual or auditory feedback (i.e., instrumental conditioning). NFT protocols exist for all frequency bands with different rationales and different clinical or non‐clinical applications (for review, please refer to Marzbani et al. 2016).

One common training protocol targets sensorimotor rhythm (SMR). The SMR is a brain wave pattern with a frequency of 12–15 Hz most prominent over the motor cortex. It is associated with calmness, improved attention, and memory (Egner and Gruzelier 2004; Vernon et al. 2003) as well as inhibition of motor activity (Sterman 2000). SMR activity is thought to reflect a thalamocortical gating mechanism that supports behavioral stillness and cognitive focus by suppressing motor output and sensory interference (Pfurtscheller and Lopes da Silva 1999; M. B. Sterman 1996). Higher SMR power prior to movement has been linked to superior accuracy in precision tasks such as air pistol shooting and darts (Cheng et al. 2015; Cheng et al. 2017). In line with that, NFT targeting SMR frequency has been shown to enhance performance in shooting sports (Gong et al. 2020), and when combined with biofeedback, it improved ice hockey shooting (Christie et al. 2020). Beyond athletes, SMR NFT has also led to improved technical performance in surgeons (Ros et al. 2009), supporting its role in fine motor skill optimization. Evidence reviewed by Jeunet et al. (2019) further indicates that SMR training not only benefits healthy individuals but also patients with neurological conditions such as ADHD, epilepsy, and stroke, where it has been associated with improvements in attention, self‐regulation, and motor recovery. SMR neurofeedback has also been shown to benefit cognition (Kober et al. 2015) and sleep in healthy adults (Hoedlmoser et al. 2008) and participants with (subclinical) insomnia (Hammer et al. 2011; Schabus et al. 2014). Additionally, SMR NFT has been suggested to benefit declarative memory consolidation due to the physiological and topographical similarities between SMR and sleep spindle activity, which have repeatedly been associated with sleep‐dependent memory consolidation (Fernandez and Lüthi 2020). Early SMR NFT studies indeed showed an effect of NFT on sleep spindle activity (Sterman et al. 1970), which has been replicated in patients with insomnia (Schabus et al. 2014). However, a follow‐up study could not find convincing effects (Schabus et al. 2017).

In general, although NFT is used in several clinical contexts (Batail et al. 2019), scientific reports on NFT effects are not conclusive and its validity has been questioned (Jeunet et al. 2019; Thibault and Raz 2017). Also, the high number of training sessions needed (Alkoby et al. 2018) and the often short‐lived nature of effects have been criticized (Marzbani et al. 2016). In light of the latter criticism, the present study explored whether NFT success can be enhanced and maybe also stabilized by combining neurofeedback with targeted memory reactivation (TMR) during sleep. For that, olfactory stimulation was used as a learning context during NFT. Several studies have shown that olfactory stimulation can enhance the consolidation of various memory representations when the presented scents were associated with the learned information (Bar et al. 2020; Diekelmann et al. 2016; Rihm et al. 2014). Olfactory cueing during slow‐wave sleep (SWS) has been shown to enhance hippocampus‐dependent memory consolidation and modulate sleep physiology, such as increasing frontal slow oscillation activity and parietal fast spindle activity (Klinzing et al. 2018; Rasch et al. 2007). Additionally, studies using functional imaging demonstrated that odor re‐exposure during sleep reactivated category‐specific memory traces which predicted post‐sleep memory performance (Shanahan et al. 2018). Targeted unilateral odor stimulation during sleep was even shown to induce hemisphere‐specific reactivation, enhancing recall for words processed in the same hemisphere, along with localized increases in spindle–slow oscillation coupling (Bar et al. 2020). A recent review by Carbone and Diekelmann (2024) further highlights the potential of TMR as a tool for enhancing learning and modulating sleep physiology.

Ultimately, a compact NFT protocol of only eight sessions was used to investigate both the possible short‐term success and, by adding a follow‐up session, the potential long‐term training effect after 10 months. SMR NFT with reactivation was compared to a random frequency protocol with reactivation as well as SMR training without reactivation. We hypothesized that NFT success and SMR power would be higher in both SMR training groups as compared to the random frequency protocol. Furthermore, NFT success should be pronounced in the SMR plus context reactivation group compared to the SMR training group without reactivation. Additionally, we expected that SMR training–particularly when combined with context reactivation–would positively affect spindle density and, consequently, memory performance. Lastly, given previous evidence that SMR NFT benefits sleep, we anticipated observable improvements in sleep parameters.

## 2 Material and Methods

### 2.1 Participants

49 participants (mean age = 24.7 years; 29 women) were included in the study. Sample size calculations were guided by prior neurofeedback studies (Hoedlmoser et al. 2008; Schabus et al. 2014), which reported an average effect size of f = 0.35 (d = 0.7) for 12–15 Hz training success and its impact on spindle activity and memory. Psychiatric or neurological disease, smoking (> 3 cigarettes per month), self‐reported impaired smell perception, photosensitivity, or a laterality quotient of > 20 in the Edinburgh Handedness Inventory (EHI; Oldfield 1971) were exclusion criteria. All participants answered the “Revised Basic Intelligence Test Scale 2” (CFT 20‐R; Weiß and Weiß 2006) and did not have clinically relevant values in the Symptom Checklist 90–Revised (SCL‐90‐R; Franke and Derogatis 2002) with T > 60 or in the Patient's Health Questionnaire depression module (PHQ‐9; Kroenke et al. 2001) with a total score > 7. Sleep Quality was assessed using the Pittsburgh Sleep Quality Index (PSQI; Buysse et al. 1989). The study was approved by the ethics committee of the Christian‐Albrechts‐University Kiel, Germany. Written informed consent was obtained from all participants.

### 2.2 Study Design

**FIGURE 1:**
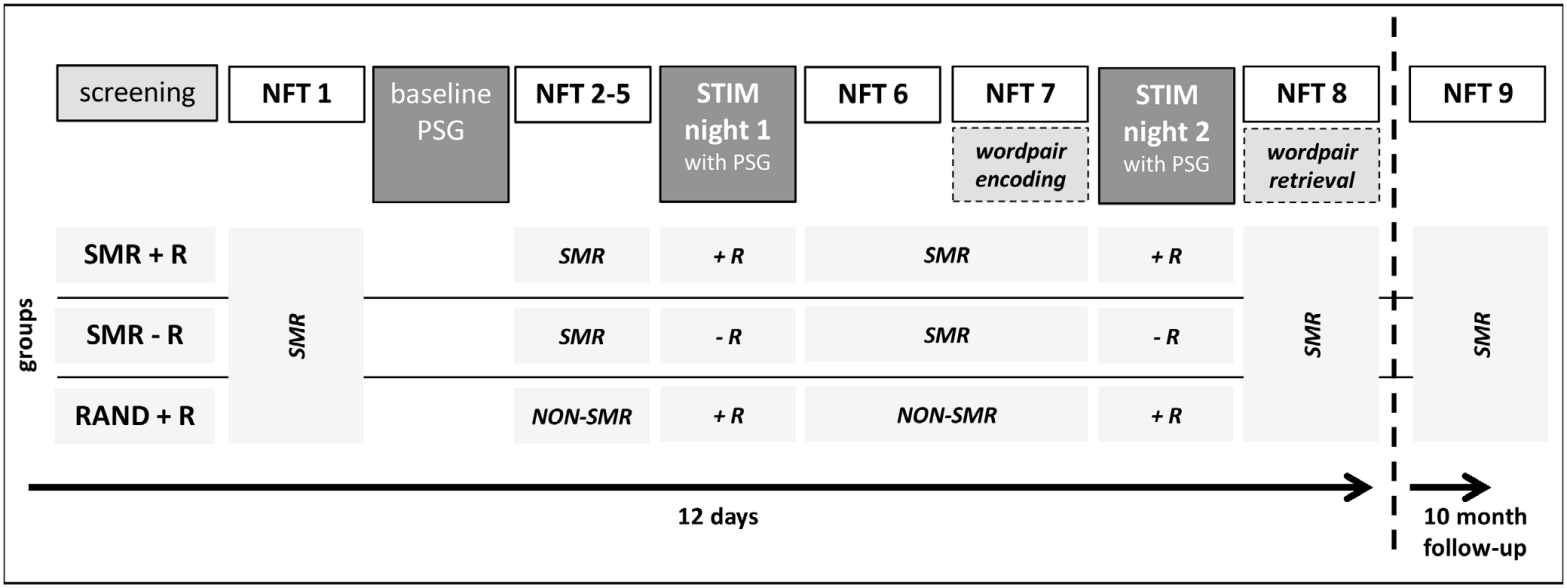
Study design. Patients were randomly assigned to one of three groups: (1) SMR neurofeedback with context reactivation (SMR + *R*), (2) SMR neurofeedback without context reactivation (SMR‐R), and (3) random frequency feedback (placebo) with context reactivation (RAND+*R*). All groups completed eight neurofeedback training (NFT) sessions. Baseline Polysomnography (PSG) was recorded after entrance examination. During stimulation night (STIM night) 1 and 2 olfactory stimulation was used to reactivate the trainings context of groups 1 and 3.

Our participants took part in a double‐blind randomized parallel group design (please refer to Figure 1).

Within 2 weeks, each group completed eight neurofeedback sessions. Participants were randomly assigned to one of three parallel groups: (1) SMR neurofeedback and reactivation (SMR + *R*; *n* = 19), (2) random frequency feedback (placebo) and reactivation (RAND+R; *n* = 19), and (3) SMR neurofeedback without reactivation (SMR‐R; *n* = 11). After sessions 1, 5, and 6, polysomnography was conducted in the sleep laboratory. Aiming to boost training success, a context was generated via olfactory stimulation during NFT in all groups. In the two reactivation groups (SMR + *R*, RAND+R), the odor was again presented for 90 min during the first sleep cycle. The SMR‐R group did also wear an oxygen mask but was not presented with the scent during the nights.

To assess potential long‐term training effect a follow‐up NFT session was conducted approximately 10 months after NFT 8.59% of original participants attended the follow‐up session (SMR + *R*: *N* = 12, RAND+*R*: *N* = 10, SMR‐R: *N* = 7).

### 2.3 Neurofeedback Training: Procedure and Analysis

**FIGURE 2:**
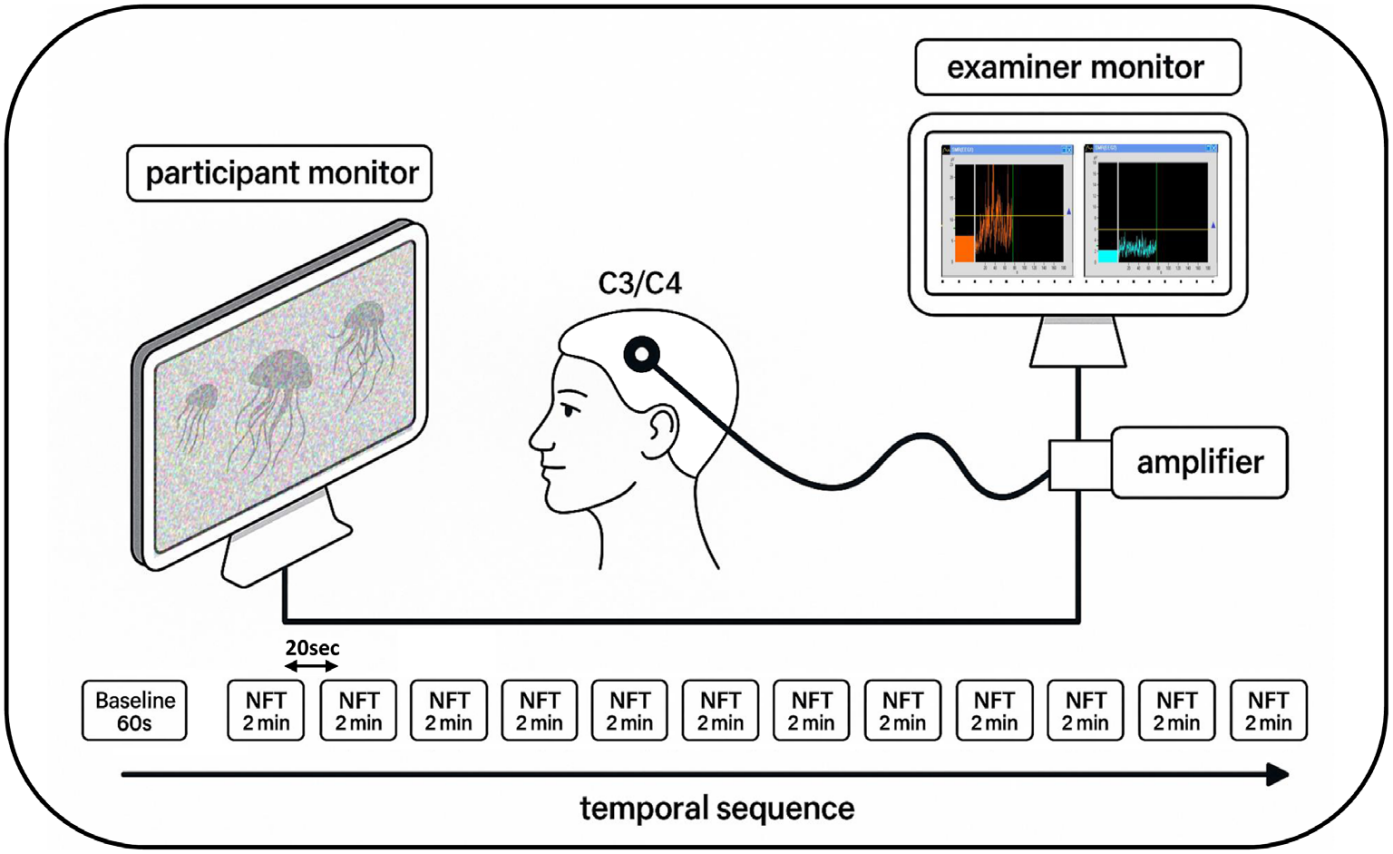
Neurofeedback setup. All participants received visual feedback in the form of a pixelated video of a jellyfish. The higher the SMR amplitude on electrodes C3 and C4 (together with low EMG frequency amplitude), the less pixelated was the video. Additional EEG derivations were recorded, but only NFT positions are shown here for clarity.

NFT was carried out using a THERA PRAX MOBILE device (neuroCare Group GmbH, Munich, Germany). In line with prior SMR NFT protocols, EEG signals for the neurofeedback protocol were derived from positions C3 and C4 (sampling rate: 256 Hz, notch filter: 50 Hz). The training also included an EMG frequency range from 20 to 40 Hz, which should control for increases in muscle tone as a source of increased EEG amplitude. During NFT, a 60 s baseline interval was followed by 12 × 2 min training intervals. Training success was calculated based on the ability to increase the respective amplitude. Feedback was provided as pixelated video (jellyfish in the ocean) which became clearer with higher amplitude. Pixelation was slowly decreased starting at –1 μV and completely inactivated at ≥ + 3 μV. Amplitude in the EMG frequency range had to stay at baseline level or below. The success rate was determined by assigning 0–1 points for each criterion within every 125 ms interval (EMG frequency ≤ baseline: 0 or 1 point; SMR frequency within baseline–1 μV to baseline + 3 μV: 0–1 points). This resulted in up to 4 points per interval. Points were summed across all intervals in each 2‐min training block, divided by the maximum possible score, and multiplied by 100 to yield a percentage. The break between training intervals was 20 s. During neurofeedback, additional EEG derivations Cz, F3, Fz, F4, P3, and P4 as well as vertical and horizontal electrooculography (EOG) were recorded. For a schematic overview of the NFT setup please refer to Figure 2.

In addition to training success rates as calculated by the NFT system, we also analyzed spectral power using Brain Vision Analyzer (Brain Products, Gilching, Germany). For that, the EEG was filtered within 0.5 to 70 Hz with a notch filter at 50 Hz. Ocular correction was conducted via independent component analysis (ICA). The signal was also visually checked for further artifacts. Both the baseline epoch at the beginning of each NFT session, as well as the 12 training epochs were segmented into 2 s segments and fast Fourier transformed (FFT). The spectral area within the SMR (12–15 Hz) band was segmented and statistically analyzed. Based on visual inspection of the resulting spectra, we decided to also conduct an exploratory analysis of the alpha band (8–12 Hz).

### 2.4 Olfactometry for Reactivation

During training intervals, participants were presented with a rose scent at regular intervals during each NFB session via oxygen mask in order to create a learning context. The scent was subsequently presented during two stimulation nights. The odor presentation was carried out using an OG001 olfactometer by Burghart, which was located in a separate room adjacent to the sleep laboratory and was connected to the oxygen mask via a Teflon tube. The rose scent consisted of a 1:25 mixture of phenylethyl alcohol (PEA, 99%) and 1,2‐propanediol.

The odor was presented at the beginning of each training block for 20 s, followed by 40 s without odor presentation (to avoid habituation). No odor was presented during the breaks between training blocks. Therefore, the scent was presented for a total of 8 min per training session.

### 2.5 Sleep Recording and Analysis

PSG was recorded according to standard AASM (American Academy of Sleep Medicine 2005) criteria, including EEG, electrocardiography (ECG), EOG, mental EMG, and leg movements using a Somnoscreen plus PSG System (Somnomedics, Randersacker, Germany). In the baseline night, also airflow, abdominal effort, and thoracic effort, as well as pulse oximetry, were recorded for diagnostic purposes (no participant suffered from sleep‐related breathing disorder).

Sleep staging was conducted by an experienced rater (who was blind to the study protocol) according to AASM criteria, resulting in the following parameters: total sleep time (TST), sleep efficiency (SE), sleep onset latency (SOL), total wake time and wake after sleep onset (WASO), and the amount of sleep stages N1, N2, N3, and REM (in minutes and percent of TST).

Sleep spindles were automatically detected in the 12–15 Hz band on C3, Cz, and C4 according to standard criteria. The procedure was adapted from previous studies (Ngo et al. 2020). Differences in spindle density (n/min) were statistically analyzed. Sleep spindles could only be analyzed in 48 participants due to technical problems.

### 2.6 Declarative Memory Test

In order to explore potential effects of successful NFT, a declarative memory test was conducted. All participants completed a word pair task on the evening of the second stimulation night. In total, 48 semantically non‐associated German word pairs were presented. All words were nouns with one to four syllables (e.g., *Arm–Kamin* [engl. arm–chimney], *Berg—Stuhl* [engl. mountain—chair], etc.). The task consisted of 4 sets of 12 pairs, which were presented sequentially. Participants were asked to memorize each pair. Two 12‐pair sets were denoted as “relevant,” whose correct retrieval was rewarded with 50 cents (“profit”), and the other two sets as “irrelevant” pairs, whose correct retrieval was not rewarded. Each 12‐pair set was presented until participants were able to correctly retrieve at least 7 pairs. During retrieval, participants were presented with the first word of the pair (cue) to remember the second word (the order of cues was pseudorandomized).

### 2.7 Statistical Analysis

Potential differences in sociodemographic data or psychopathology were analyzed using one‐way ANOVAs for factor GROUP (SMR + *R*, SMR‐R, and RAND + R) with dependent variables age, IQ, and total scores of PHQ‐9, SCL‐90, and PSQI. Sex distribution was compared between groups by chi‐squared test.

In order to compare the different NFT protocols, linear mixed models with fixed factors GROUP (SMR + reactivation [SMR + *R*], SMR‐reactivation [SMR‐R], random frequency + reactivation [RAND+*R*]) as well as NFT SESSION (NFT 1, NFT 8, and follow‐up) were analyzed. Participants were included as a random factor. Neurofeedback success (provided by internal calculations of the THERA PRAX device), SMR power (μV*Hz), and alpha power (μV*Hz) were used as dependent variables. Effects were alike on both hemispheres but slightly pronounced on the left side; wherefore, only C3 results will be reported.

To analyze potential differences in objective sleep parameters between groups from baseline and second stimulation night, models for fixed effects of GROUP (SMR + *R* vs. SMR‐R vs. RAND+*R*) and NIGHT (baseline, stimulation night 2) were calculated for dependent variables sleep efficiency (SE), sleep onset latency (SOL), total wake time, wake after sleep onset (WASO), and changes in the percentage of stages N1, N2, N3, and REM and sleep spindle density.

Each model was calculated twice: (1) without interactions between fixed effects (primary model) and (2) with interactions between fixed effects (secondary model). Comparing these models allowed us to assess whether the added complexity of the interaction improved model fit and whether main effects were robust across model specifications. Models were compared with respect to changes in the Akaike information criterion (primary model: AIC1; secondary model: AIC2). ΔAIC < 2 indicates little difference, 4–7 moderate improvement, and > 10 strong support for the model with the lower AIC. For fixed effects, we report F‐values along with degrees of freedom (df) as well as estimated marginal means (M), standard error of mean (SEM), and *p*‐values. Interactions were post hoc evaluated using t‐tests. The significance level was *p* = 0.05 (two‐tailed); *p*‐values < 0.10 are considered trends. Significance levels for post hoc tests were corrected for multiple comparisons according to Benjamini and Hochberg (2000) and denoted as p_corr_.

Furthermore, a one‐way ANOVA was calculated for GROUP (SMR + *R*, SMR‐R, and RAND+R) with dependent variable overnight memory change (OMC) in the word pair task. Normal distribution was tested via the Kolmogorov–Smirnov test for each group, and equality of variances was tested via Levene's test. Both assumptions were always met in the respective data (*p* > 0.05).

Effect sizes (partial η^2^ [ηp^2^]) for main effects and interactions were estimated based on F‐values and degrees of freedom. For the post hoc comparisons, we report Cohen's d as effect size.

Lastly, a Pearson correlation between spindle density and OMC in the memory task was calculated.

All analyses were calculated using SPSS version 28.01.1.

## 3 Results

### 3.1 Sociodemographic Data and Psychopathology

The three groups did not differ in sex (*p* = 0.94), age, or IQ (F_2,46_ < 0.68, *p* > 0.51). Group differences regarding PHQ‐9 total score emerged (F_2,46_ = 4.48, *p* = 0.02; SMR + *R* < SMR‐R: *p* = 0.02, p_corr_ = 0.03; SMR + *R* < RAND+*R: p* = 0.004, p_corr_ = 0.006; SMR‐R vs. RAND+*R*: *p* = 0.74, p_corr_ = 0.74). Accordingly, the SCL‐90 subscale depression showed the same tendency (F_2,46_ = 3.09, *p* = 0.06). The respective scores were, however, in all groups below the clinical cut‐off. PSQI total scores did not differ between groups (F_2,38_ = 0.32, *p* = 0.73). Sample characteristics are displayed in Table 1.

**TABLE 1:** Sample characteristics. The three groups did not differ in age, sex, or IQ.

	Groups		
	SMR [+reactivation] Mean (SD)	SMR [−reactivation] Mean (SD)	Random [+reactivation] Mean (SD)
Age	24.89 (2.51)	23.91 (2.81)	25.00 (2.60)
IQ (CFT)	118.63 (12.37)	114.36 (11.09)	118.11 (11.96)
SCL‐90 GSI	30.37 (2.57)	29.82 (2.52)	30.11 (2.54)
PHQ‐9	2.21 (1.69)	4.64 (3.72)	4.26 (2.38)
PSQI	3.80 (1.66)	4.27 (1.85)	3.80 (1.57)

Abbreviations: GSI, global severity index; SD, standard deviation.

### 3.2 Evaluation of NFT Effects

#### 3.2.1 NFT Success

**FIGURE 3:**
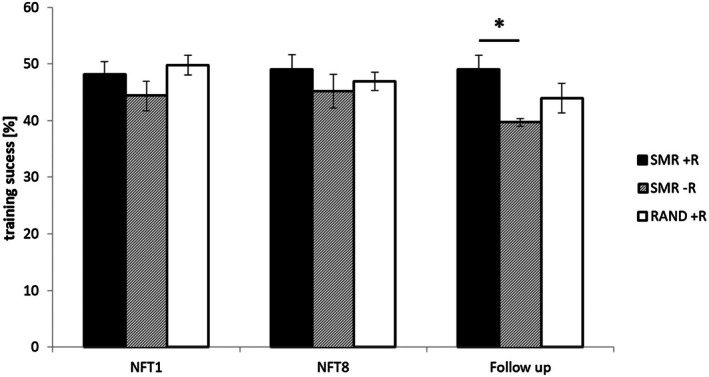
Neurofeedback success rates from NFT 1 to follow‐up. During the follow‐up neurofeedback session the SMR + *R* (i.e., SMR with reactivation) group showed a higher success rate than the SMR‐R group (i.e., SMR without context reactivation).

The model with fixed factors NFT SESSION (1, 8, and follow‐up) and GROUP (SMR + *R*, SMR‐R, and RAND+*R*) with the dependent variable training success [%] showed no effect of NFT SESSION (F_2,41.01_ = 1.34, *p* = 0.27, ηp^2^ = 0.061), but a significant effect of GROUP (F_2,41.44_ = 3.32, *p* < 0.05, ηp^2^ = 0.138). The secondary model did again show a main effect of GROUP (F_2, 41.48_ = 3.17, *p* = 0.05, ηp^2^ = 0.13) but not for NFT SESSION (F_2,38.51_ = 1.59, *p* = 0.22, ηp^2^ = 0.08). According to pairwise comparisons, the SMR + *R* group (M = 48.74, SEM = 1.34) had higher success rates than the SMR‐R group (M = 43.07, SEM = 1.76) over all training occasions (*p* = 0.01, p_corr_ = 0.04, d = 0.97). Despite the better model fit (AIC2–AIC1 = −21.45), the interaction between NFT SESSION and GROUP was not significant (F_4,38.53_ = 0.87, *p* = 0.49, ηp^2^ = 0.083), suggesting no strong evidence for an interaction effect. To further explore the data, pairwise comparisons were conducted and revealed higher success rates in the SMR + *R* than in the SMR‐R group during follow‐up NFT session only (T_12.71_ = 3.78, *p* < 0.01, p_corr_ = 0.02, d = 1.43, please refer to Figure 3), but neither between SMR + *R* and RAND+*R* (T_20_ = 1.44, *p* = 0.16, p_corr_ = 0.36, d = 0.47), nor RAND+*R* and SMR‐R (T_10.18_ = 1.58, *p* = 0.14, p_corr_ = 0.36, d = 0.60). During NFT 1 and 8, no group differences were evident in the pairwise comparisons.

#### 3.2.2 Effects on SMR Power

The primary model with fixed factors NFT SESSION (1, 8, and follow‐up) and GROUP (SMR + *R*, RAND+R and SMR‐R) with the dependent variable C3 SMR amplitude showed neither an effect of NFT SESSION (F_2,38.31_ = 0.31, *p* = 0.73, ηp^2^ = 0.016) nor GROUP (F_2,45.47_ = 1.45, *p* = 0.25, ηp^2^ = 0.060). The secondary model did not yield a better model fit (AIC2–AIC1 = 0.21) and again showed no main effect for NFT SESSION (F_2, 35.96_ = 0.18, *p* = 0.84, ηp^2^ = 0.01) or GROUP (F_2, 44.56_ = 1.34, *p* = 0.27, ηp^2^ = 0.06) and no interaction between NFT SESSION and GROUP (F_4,36.10_ = 0.663, *p* = 0.622, ηp^2^ = 0.068).

#### 3.2.3 Effects on Alpha Power

**FIGURE 4:**
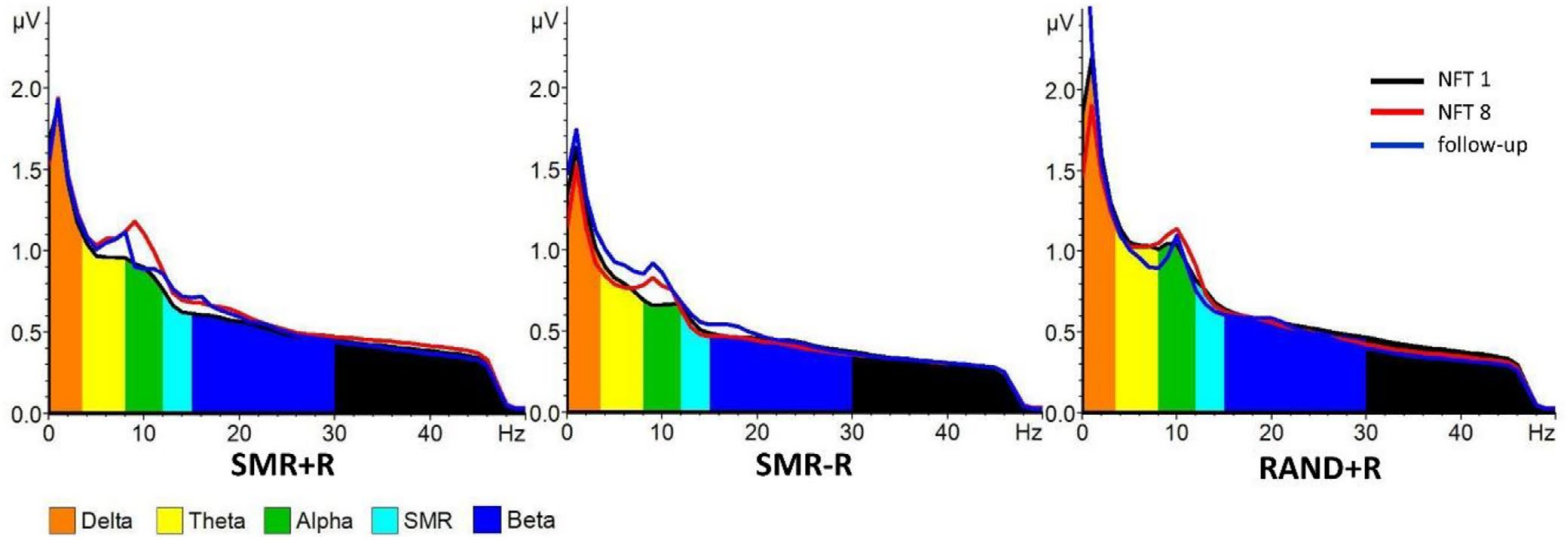
EEG spectra during NFT 1, NFT 8, and follow‐up for all three training protocols. In all NFT protocols, alpha power increased from NFT 1 to NFT 8.

The primary model with fixed factors NFT SESSION (1, 8, and follow‐up) and GROUP (SMR + *R*, RAND+R and SMR‐R) with the dependent variable C3 alpha amplitude showed a tendency for a fixed effect of NFT SESSION (F_2,34.25_ = 2.70, *p* = 0.08, ηp^2^ = 0.136), but not for GROUP (F_2,45.84_ = 2.01, *p* = 0.15, ηp^2^ = 0.081). Post hoc comparisons revealed by tendency a higher alpha power during NFT 8 (M = 3.97, SEM = 0.27) as compared to NFT 1 (M = 3.45, SEM = 0.28; *p* = 0.03, p_corr_ = 0.08, d = 0.27) irrespective of group (please refer to Figure 4). The secondary model only slightly improved the model fit (AIC2–AIC1 = −3.65) but showed no main effect of NFT SESSION (F_2, 32.02_ = 2.40, *p* = 0.11, ηp^2^ = 0.13) or GROUP (F_2, 43.50_ = 1.69, *p* = 0.20, ηp^2^ = 0.07) and no interaction between NFT SESSION and GROUP (F_4,32.18_ = 0.36, *p* = 0.84, ηp^2^ = 0.043).

### 3.3 Effects on Sleep and Memory

#### 3.3.1 Spindle Density

The primary model with fixed factors NIGHT (baseline, stimulation night 2) and GROUP (SMR + *R*, SMR‐R, and RAND+*R*) with the dependent variable C3 spindle amplitude did neither show an effect of NIGHT (F_1,47_ = 0.03, *p* = 0.87, ηp^2^ = 0.001) or GROUP (F_2,45_ = 0.71, *p* = 0.50, ηp^2^ = 0.031). The secondary model yielded slightly worse model fit (AIC2–AIC1 = 3.07) and again showed no main effect for GROUP (F_2, 45_ = 0.48, *p* = 0.63, ηp^2^ = 0.02) or NIGHT (F_1, 45_ = 0.02, *p* = 0.88, ηp^2^ < 0.01) and no interaction between NFT SESSION and GROUP (F_2,45_ = 1.06, *p* = 0.35, ηp^2^ = 0.045).

#### 3.3.2 Effects of Training Protocol and Reactivation on Memory Tasks

**FIGURE 5:**
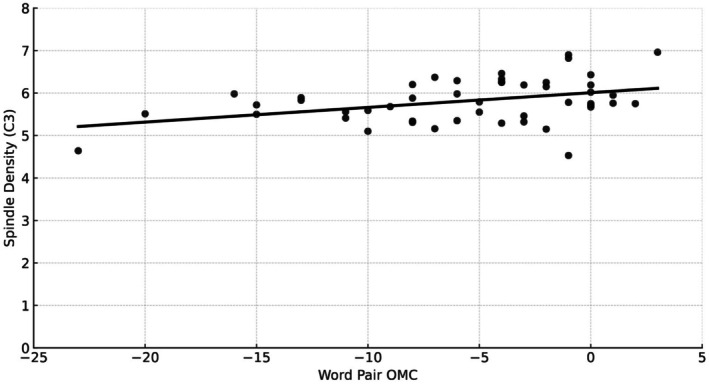
Correlation between spindle density at position C3 with overnight memory change (OMC) in the word pair task over all three groups (*r* = 0.40, *p* < 0.01).

The ANOVA for GROUP (SMR + *R*, SMR‐R, and RAND+R) with the dependent variable Overnight Memory Change (OMC) showed no differences between the three groups (F_2,46_ = 0.47, *p* = 0.63, ηp^2^ = 0.02). However, the OMC over all groups correlated with spindle density in the subsequent night (i.e., stimulation night 2, *r* = 0.40, *p* < 0.01; please refer to Figure 5).

#### 3.3.3 Objective Sleep Parameters

The first model for the dependent variable sleep efficiency (%) did show a main effect of NIGHT (F_1,49.08_ = 8.37, *p* < 0.01, ηp^2^ = 0.15), indicating higher sleep efficiency during the second night (M = 90.99, SEM = 0.62) as compared to the baseline night (M = 86.84, SEM = 1.54). No main effect was found for GROUP (F_2,46.56_ = 1.33, *p* = 0.27, ηp^2^ = 0.05). The extended model yielded a better model fit (AIC2–AIC1 = −15.01) and again showed a main effect of NIGHT (F_1,47.23_ = 10.58, *p* < 0.01, ηp^2^ = 0.18) and a significant interaction between GROUP and NIGHT (F_2,47.35_ = 3.39, *p* = 0.04, ηp^2^ = 0.13). Again, no main effect was found for GROUP (F_2, 47.25_ = 1.11, *p* = 0.34, ηp^2^ = 0.04). Post hoc t‐tests showed by tendency higher sleep efficiency in the second compared to the baseline night only in the SMR groups (SMR + *R*: M = 90.71, SEM = 0.90 vs. M = 83.87, SEM = 3.18, T_18_ = −2.25, *p* = 0.04, p_corr_ = 0.07, d = 0.52; SMR‐R: M = 93.04, SEM = 0.65 vs. M = 85.68, SEM = 3.28, T_10_ = −2.23, *p* = 0.05, p_corr_ = 0.07, d = 0.67), but not for the RAND+*R* group (M = 90.10, SEM = 1.23 vs. M = 90.49, SEM = 1.23, T_18_ = 0.39, *p* = 0.70, p_corr_ = 0.70, d = 0.09).

The first model for the dependent variable sleep onset latency (min) showed no significant main effect of GROUP (F_2, 47.37_ = 0.16, *p* = 0.86, ηp^2^ = 0.01) or of NIGHT (F_1, 49.26_ = 0.11, *p* = 0.74, ηp^2^ = 0.00). The extended model yielded a better model fit (AIC2–AIC1 = −15.38). It showed no main effect for GROUP (F_2, 47.49_ = 0.49, *p* = 0.62, ηp^2^ = 0.02) or NIGHT (F_1, 47.63_ = 0.26, *p* = 0.61, ηp^2^ = 0.01), but a trend toward an interaction between GROUP and NIGHT (F_2,47.48_ = 2.42, *p* = 0.10, ηp^2^ = 0.09). Post hoc t‐tests showed by tendency a longer sleep onset latency in the second compared to the first night in the RAND+*R* group (M = 16.41, SEM = 3.03 vs. M = 10.47, SEM = 2.49, T_18_ = –2.36, *p* = 0.030, p_corr_ = 0.09, d = 0.54).

**FIGURE 6:**
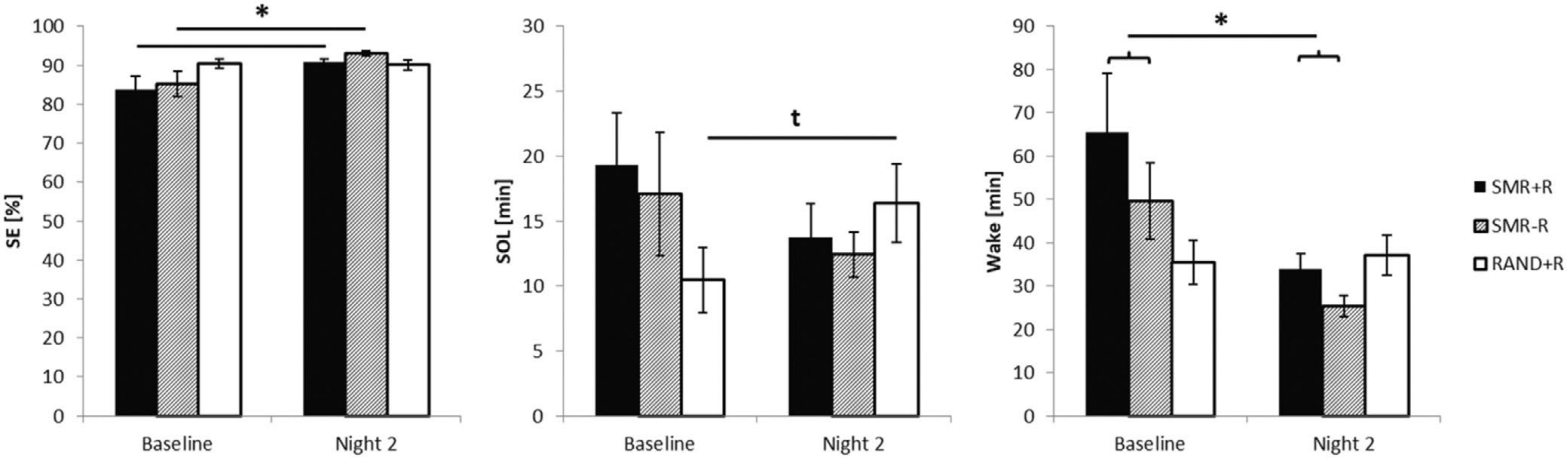
Changes in objective sleep parameters from baseline night to second stimulation night (Night 2, after NFT 7). Both SMR training groups showed an increase in sleep efficiency (SE) and decrease in total wake time. The random NFT group had by tendency higher sleep onset (SOL) latency in the second compared to the baseline night. *significant post hoc test, t statistical trend.

The first model for dependent variable total wake time (min) showed a main effect of NIGHT (F_1,48.00_ = 6.36, *p* = 0.02, ηp^2^ = 0.12) indicating shorter wake time during the second as compared to baseline but no effect for GROUP (F_2,46.51_ = 1.06, *p* = 0.36, ηp^2^ = 0.04). The extended model yielded a better model fit (AIC2–AIC1 = −19.80). It showed a main effect of NIGHT (F_1, 46_ = 7.13, *p* = 0.01, ηp^2^ = 0.13), no main effect of GROUP (F_2, 46_ = 1.19, *p* = 0.31, ηp^2^ = 0.05) as well as a trend toward an interaction between GROUP and NIGHT (F_2,46_ = 2.66, *p* = 0.08, ηp^2^ = 0.10). Wake time was shorter in the second stimulation compared to the baseline night in both SMR groups, post hoc t‐tests did, however, not reach significance when corrected for multiple comparisons. Since the effect was, however, similar (please refer to Figure 6) in both SMR groups, a comparison of second stimulation night against baseline for both groups together revealed a significant effect (M = 30.81, SEM = 2.98 vs. M = 59.72, SEM = 10.58, T_29_ = 2.84, *p* = 0.008, p_corr_ = 0.02, d = 0.52).

## 4 Discussion

The main aim of the presented study was to improve SMR neurofeedback training effects by cue‐dependent memory reactivation during sleep in a compact NFT protocol. For this, we compared SMR training with olfactory reactivation during sleep against a random frequency protocol with reactivation and a SMR protocol without reactivation. Neither one of our groups showed an increase in relative training success nor a significant increase in SMR power over the course of eight NFT sessions. During follow‐up NFT, however, success rates were higher in the SMR training group with as compared to without context reactivation. Over all training groups, we did see a trend for an elevation of alpha power over time. Although we did not find a significant increase in SMR training success or amplitude, only the SMR training groups showed an increase in sleep efficiency in the last night as compared to the baseline polysomnography. Furthermore, the random frequency group showed an increase in sleep onset latency, while in the SMR groups, SOL numerically decreased. In both SMR groups, but not in the random protocol, total wake time was shorter in the last as compared to the baseline night. Concerning memory performance and spindle density, no differences between training groups were evident in our data.

As mentioned before, our main intention was to boost training success via context reactivation during sleep. Our analysis showed that reactivation of SMR training context during sleep did not immediately boost NFT success. Exploratory post hoc comparisons suggested that reactivation might have the potential to prevent a deterioration of NFT success and could help to stabilize long‐term training strategies. However, the follow‐up effect is limited by the fact that only 59% of participants attended the follow‐up appointment. Nevertheless, it would be interesting to test whether more nights in the sleep lab for context reactivation earlier in the training period could increase (long‐term) effects.

In general, SMR neurofeedback studies have yielded heterogeneous results. While some studies showed effects on SMR amplitude over the course of training, others have not (for review, please refer to Ribeiro et al. 2023). In our data, neither training success nor SMR amplitude showed a significant increase over time. However, changes in objective sleep parameters did (by tendency) differ between groups. Both SMR groups but not the random frequency group showed an increase in sleep efficiency. In contrast, the random frequency group had longer SOL at the end of the training, while SOL in the SMR groups numerically decreased. Total wake time was in both SMR groups shorter during the second stimulation night as compared to baseline. Effects of SMR NFT on sleep onset latency have been reported before, both in healthy (Hoedlmoser et al. 2008) and in patients with ADHD (age 6–53 yrs.; Arns et al. 2014).

As a secondary finding, we saw a trend toward an increase of alpha amplitude from session 1 to 8 irrespective of the training protocol. The alpha band is the dominant frequency in the EEG during wakefulness. During relaxed wakefulness and reduced sensory processing (especially with eyes closed), an increase in alpha amplitude can be observed. Both alpha synchronization and desynchronization have been associated with different aspects of cognitive processing (Klimesch 2012; Pfurtscheller 2001). It has been shown that localized increases in alpha power inhibit the processing of irrelevant information, thereby enhancing the efficiency of neural networks involved in task‐relevant processing (Händel et al. 2011). We argue that the training setting with quiet alertness could have affected alpha amplitude independent of the specific frequency being trained. As compared to other studies mentioned before, our NFT consisted of longer training periods, which might have allowed for entering a focused attentive state and could have specifically increased alpha activity similar, perhaps, to meditative practices for which an increase in alpha amplitude has repeatedly been reported (Fell et al. 2010; Lagopoulos et al. 2009). Visual inspection of the spectra pointed toward a stronger effect in the SMR groups, yet statistical data analysis did not reveal a significant interaction. Please keep in mind, however, that all three groups trained SMR during sessions 1, 8, and follow‐up, which could have decreased potential interaction effects. To fully answer the question of whether specific frequency protocols or at least the training of a constant frequency is superior to random NFT, comparing different constants against random frequency protocols and investigating their respective effects in future studies would be necessary.

Concerning sleep microstructure, spindle density was not higher at the end of the training protocol as compared to the baseline night in none of the three groups. Also, no differences were found for memory performance between training groups. Similar results have been reported for patients with primary insomnia (Schabus et al. 2017). As a secondary finding, we replicated an often reported correlation between overnight memory change and spindle density during NREM sleep (Fernandez and Lüthi 2020).

Despite the methodological rigor of this study, several limitations must be acknowledged. First, the small sample size, particularly in the SMR‐R group, may have limited statistical power and increased the risk of type II errors, reducing the ability to detect subtle group differences or interactions. Additionally, as mentioned before, only 59% of participants returned for the follow‐up session, limiting conclusions about long‐term effects. Furthermore, for a fully balanced design, future studies should also include also a group training random frequency bands without context reactivation. And lastly, in clinical settings, usually more NFT sessions are planned. Although our design choices were deliberate, a higher number of sessions might have helped to gain stronger results.

## 5 Conclusion

In conclusion, our data showed that NFT seems to have increased alpha band power, and SMR NFT in particular seemed to have benefitted sleep. An increase in alpha power could indicate that the neurofeedback training helps participants achieve a state of enhanced relaxation and focused internal calm. This is particularly relevant if the goal of the training is to reduce stress or improve attentional control. The observed improvements in sleep efficiency and reductions in wake time in both SMR groups align with previous studies and support the potential of NFT as a non‐pharmacological intervention to promote sleep continuity. This is especially relevant given the increasing demand for behavioral and neurocognitive sleep therapies with minimal side effects. Although contextual reactivation via olfactory stimulation did not enhance immediate NFT outcomes, it may contribute to the stabilization of training effects over time. Using olfactory cues for memory reactivation during sleep represents a novel strategy for consolidating NFT‐related learning, and may inform future approaches to enhancing sleep‐dependent memory processes. At the end, for disentangling the specific effects of NFT, a comparison of several different constant and random frequency protocols in a randomized double‐blind manner would be vital for future NFT research and its clinical applications. With regard to the question of whether NFT with context reactivation could be superior to standard NFT, more NFT sessions and more reactivation nights in the sleep laboratory would be necessary. So far, different forms of targeted memory reactivation (TMR) have yielded promising results (Carbone and Diekelmann 2024). As an alternative to olfactory context reactivation, the use of acoustic stimuli could boost NFT success more directly by displaying the same sound during training as positive feedback (reinforcing only successful training) and again for targeted memory consolidation during sleep. Displaying auditory feedback both during training and during sleep for reactivation could be simpler yet more effective. In general, the possibility to reactivate memory context and significantly boost learning during sleep would be a promising tool to augment also other therapeutic effects (e.g., psychotherapy). This may be especially valuable for populations with subclinical sleep disturbances or cognitive fatigue, for whom more intensive therapeutic interventions may not yet be warranted.

## Author Contributions

**Julia Lechinger:** writing – original draft, formal analysis, visualization, methodology, software, writing – review and editing. **Catarina Newe:** methodology, conceptualization, investigation, writing – original draft, writing – review and editing, data curation, project administration, software, formal analysis. **Svenja Nadler:** project administration, data curation, investigation, software. **Marlene Schubert:** data curation, project administration, software, investigation. **Hong‐Viet V. Ngo:** methodology, formal analysis. **Robert Göder:** resources, writing – review and editing. **Alexander Prehn‐Kristensen:** conceptualization, supervision, project administration, writing – review and editing, funding acquisition, methodology, investigation, resources, validation.

## Ethics Statement

The study was approved by the ethics committee of the Christian‐Albrechts‐University Kiel, Germany.

## Consent

Written informed consent was obtained from all participants.

## Conflicts of Interest

The authors declare no conflicts of interest.

## Supporting information


**Figure S1** Sleep efficiency across nights by group. Mean sleep efficiency (±standard error) at Baseline and Night 2 is shown for each of the three experimental groups: SMR + *R*, SMR‐R, and RAND+*R*. Individual participant trajectories are depicted as gray lines, highlighting intra‐individual changes over time. Group means are indicated with bold black lines and error bars.
**Figure S2** Sleep onset latency (SOL) across two nights (Baseline and Night 2), separated by experimental group. Individual trajectories are shown in color, with each line representing one participant. Group means are indicated by black lines with error bars showing the standard error of the mean (±SE). Overall, SOL appears to decrease from Baseline to Night 2 only in the two SMR groups. In contrast, mean SOL increased in the random frequency protocol.
**Figure S3** Alpha activity over time by group. Mean alpha (8–12 Hz) area (μV*Hz ± standard error) is shown across three measurement time points: NFT 1 (start of neurofeedback training), NFT 8 (end of training), and follow‐up. Separate subplots display data for the three experimental groups: SMR + *R*, SMR‐R, and RAND+*R*. Colored lines represent individual participant trajectories, illustrating within‐subject changes across sessions. Group means with standard errors are plotted in bold.

## Data Availability

The data that support the findings of this study are available on request from the corresponding author. The data are not publicly available due to privacy or ethical restrictions.
