# Supplementary material for: Effects of Olfactory Learning Context Reactivation During Sleep on Training Success in a Double‐Blind Randomized Controlled Sensorimotor Rhythm Neurofeedback Protocol: A Pilot Study
Source: Psychophysiology. 2025 Oct 8;62(10):e70154. doi: 10.1111/psyp.70154 (PMC12506616; doi:10.1111/psyp.70154)
Supplement: Supplementary file 1 — Figure S1 Sleep efficiency across nights by group. Mean sleep efficiency (±standard error) at Baseline and Night 2 is shown for each of the three experimental groups: SMR + R, SMR‐R, and RAND+R. Individual participant trajectories are depicted as gray lines, highlighting intra‐individual changes over time. Group means are indicated with bold black lines and error bars. Figure S2 Sleep onset latency (SOL) across two nights (Baseline and Night 2), separated by experimental group. Individual trajectories are shown in color, with each line representing one participant. Group means are indicated by black lines with error bars showing the standard error of the mean (±SE). Overall, SOL appears to decrease from Baseline to Night 2 only in the two SMR groups. In contrast, mean SOL increased in the random frequency protocol. Figure S3 Alpha activity over time by group. Mean alpha (8–12 Hz) area (μV*Hz ± standard error) is shown across three measurement time points: NFT 1 (start of neurofeedback training), NFT 8 (end of training), and follow‐up. Separate subplots display data for the three experimental groups: SMR + R, SMR‐R, and RAND+R. Colored lines represent individual participant trajectories, illustrating within‐subject changes across sessions. Group means with standard errors are plotted in bold. [file PSYP-62-e70154-s001.docx]

**Supplementary material**


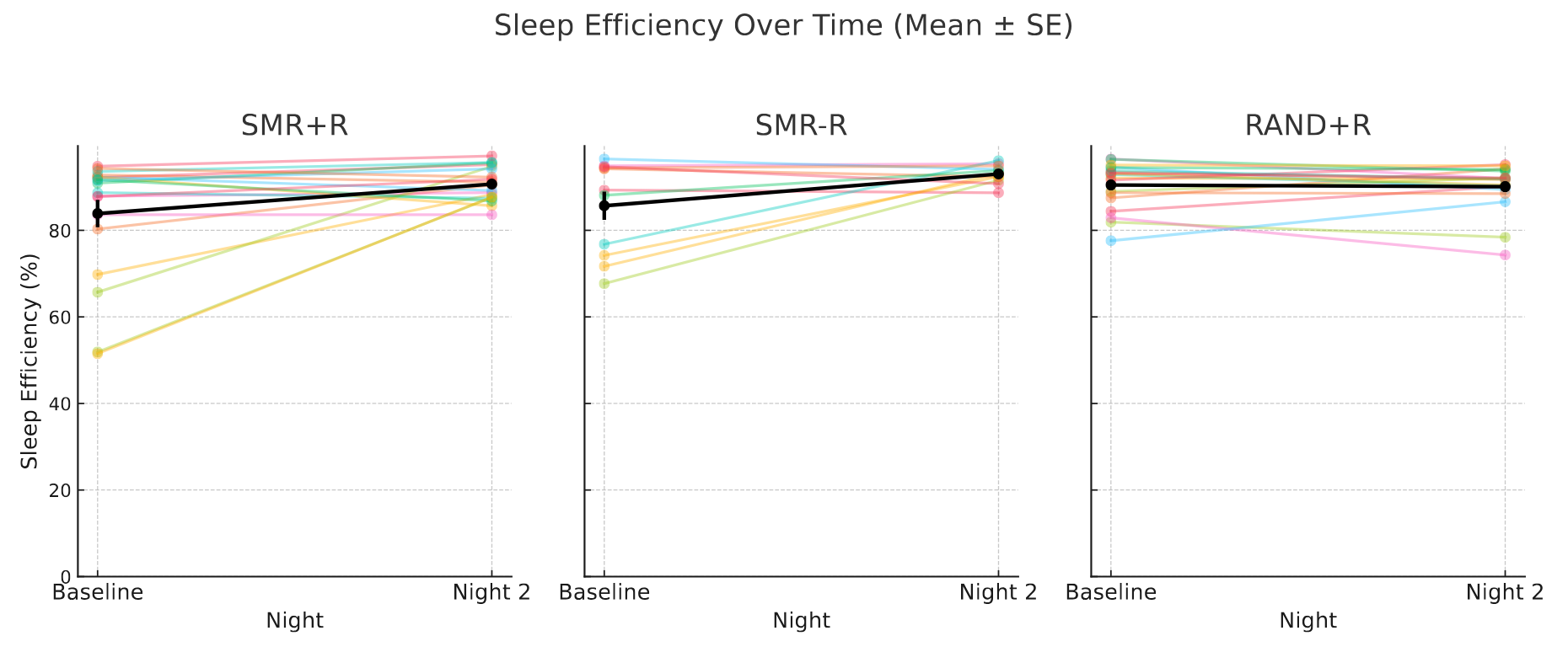


**Figure S1.** Sleep efficiency across nights by group. Mean sleep efficiency (± standard error) at Baseline and Night 2 is shown for each of the three experimental groups: SMR+R, SMR-R, and RAND+R. Individual participant trajectories are depicted as gray lines, highlighting intra-individual changes over time. Group means are indicated with bold black lines and error bars.


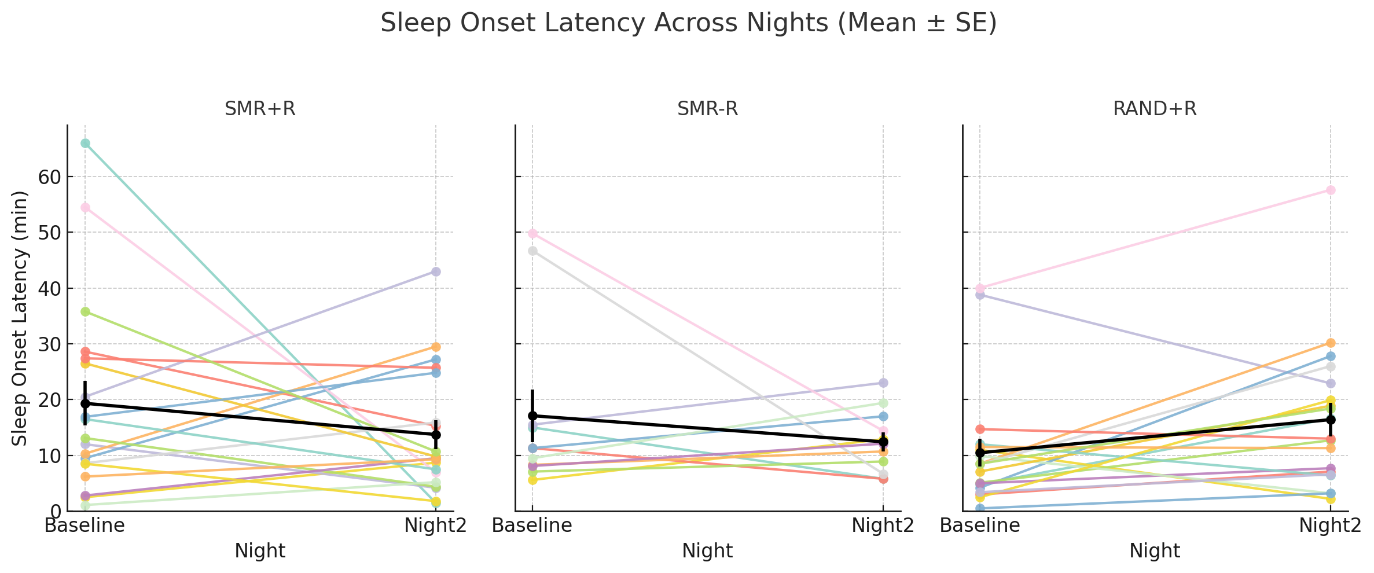


**Figure S2**. Sleep onset latency (SOL) across two nights (Baseline and Night 2), separated by experimental group. Individual trajectories are shown in color, with each line representing one participant. Group means are indicated by black lines with error bars showing the standard error of the mean (±SE). Overall, SOL appears to decrease from Baseline to Night 2 only in the two SMR groups. In contrast, mean SOL increased in the random frequency protocol.

**
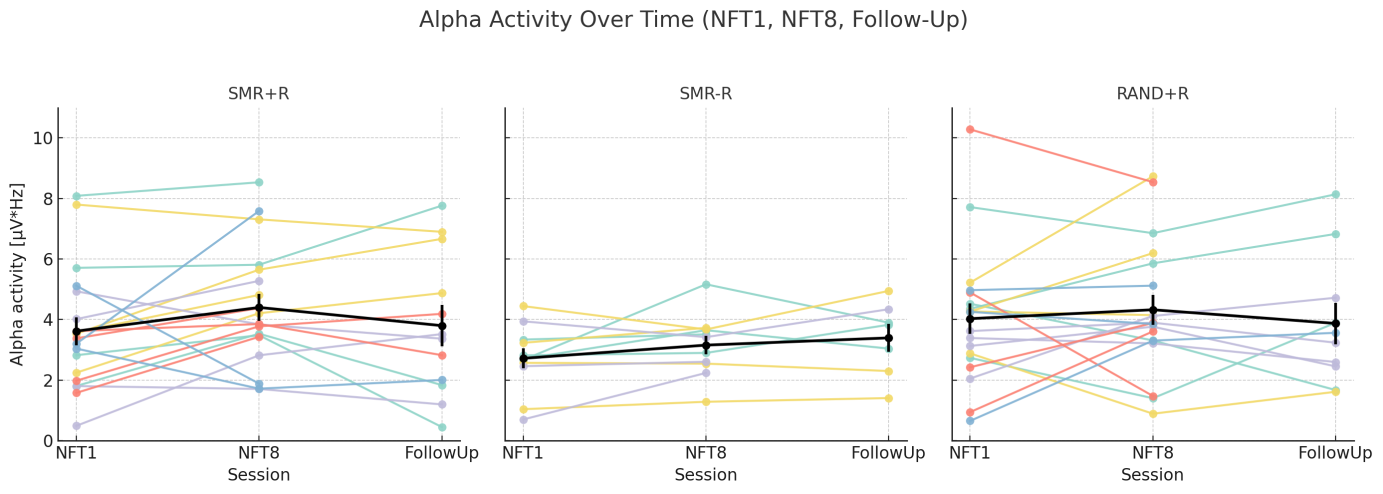
**

**Figure S3.** Alpha activity over time by group. Mean alpha (8-12Hz) area (µV*Hz ± standard error) is shown across three measurement time points: NFT 1 (start of neurofeedback training), NFT 8 (end of training), and Follow-up. Separate subplots display data for the three experimental groups: SMR+R, SMR-R, and RAND+R. Colored lines represent individual participant trajectories, illustrating within-subject changes across sessions. Group means with standard errors are plotted in bold.
